# Predatory Earwigs are Attracted by Herbivore-Induced Plant Volatiles Linked with Plant Growth-Promoting Rhizobacteria

**DOI:** 10.3390/insects11050271

**Published:** 2020-04-29

**Authors:** Kim Bell, Natalia Naranjo-Guevara, Rafaela C. dos Santos, Richard Meadow, José M. S. Bento

**Affiliations:** 1 Department of Plant Sciences, Faculty of Biosciences, Norwegian University of Life Sciences, 1433 Ås, Norway; kimfantastico@gmail.com (K.B.); richard.meadow@nmbu.no (R.M.); 2 Department of Entomology and Acarology, Luiz de Queiroz College of Agriculture, University of São Paulo, Piracicaba, São Paulo State 13418-900, Brazil; nnaranjoguevara@gmail.com (N.N.-G.); santosrc@usp.br (R.C.d.S.); 3 Fontys International Business School Venlo, Fontys University of Applied Sciences, 5912 Venlo, The Netherlands

**Keywords:** *Bacillus amyloliquefaciens* GB03, Brassicaceae, insect–plant–microbe interactions, olfactory behavior, soil-borne bacteria

## Abstract

Plant-associated microbes may induce plant defenses against herbivores. Plants, in turn, can attract natural enemies, such as predators, using herbivore-induced plant volatiles. Intricate communication occurs between microorganisms, plants, and insects. Given that many aspects related to mechanisms involved in this symbiotic system remain unknown, we evaluated how beneficial soil-borne microorganisms can affect the interactions between plants, herbivores, and natural enemies. For this study, we established a multitrophic system composed of the predatory earwig *Doru luteipes* (Dermaptera: Forficulidae), arugula (*Eruca sativa*, Brassicaceae) as the host plant, *Plutella xylostella* (Lepidoptera: Plutellidae) larvae as a specialist herbivore, *Spodoptera frugiperda* (Lepidoptera: Noctuidae) larvae as a generalist herbivore, and *Bacillus* *amyloliquefaciens* as the plant growth-promoting rhizobacteria (PGPR), in a series of nocturnal olfactometry experiments. By assessing earwig preference towards herbivore-induced and PGPR-inoculated plants in different combinations, we showed that the interaction between rhizobacteria, plants, and herbivores can affect the predatory earwig’s behavior. Furthermore, we observed a synergistic effect in which earwigs were attracted by plants that presented as PGPR inoculated and herbivore damaged, for both specialist and generalist herbivores. Our findings help fill the important knowledge gap regarding multitrophic interactions and should provide useful guidelines for their application to agricultural fields.

## 1. Introduction

In natural environments, plants are exposed to various types of attacks, including those by herbivorous arthropods. To defend themselves, they have developed complex mechanisms, such as constitutive and induced defenses. Constitutively, plants can affect herbivores through physical barriers, as lignified cell walls, trichomes, and callose deposits [1], or by biochemical pathways as well as the synthesis of secondary metabolites [2]. Plants can be induced to express direct and indirect defenses in response to herbivore attacks. Directly, plants produce toxins or digestion inhibitors [3]. Indirectly, plants use herbivore-induced plant volatiles (HIPVs), which act as odor cues to attract natural enemies of herbivores, i.e., predators or parasitoids, which use these chemical cues to search for prey or hosts [4,5,6,7,8,9,10].

Different herbivore species elicit different volatile compositions [5,6,11]. Thus, specialist and generalist herbivorous insects can interact with plants in different ways and therefore, trigger different responses against attack [12,13,14]. This could be due to the activation of different plant secondary metabolic pathways [5]. Likewise, natural enemies can discriminate between the attack by different herbivore species [15,16]. It has been shown, for example, that parasitoids of *Diabrotica balteata* and *D. virgifera virgifera* (Chrysomelidae: Coleoptera) preferred roots attacked by specialists over roots damaged by generalist herbivores in maize plants [17]. Similarly, it was observed that the specialist parasitoid *Cotesia rubecula* (Hymenoptera: Braconidae) preferred *Arabidopsis thaliana* plants damaged by chewing insects to those damaged by phloem feeders [18].

Plant-associated microbes can also influence indirect plant defenses and the recruitment of natural enemies [19,20]. Plant-beneficial soil-borne bacteria are known to induce plant defenses [21] and may cause interactions with higher trophic levels [22]. These bacteria associations have also been shown to produce volatile organic compounds (VOCs), which enhance plant fitness and growth [23,24], and have an effect on the attraction of herbivore antagonists, such as predators and parasitoids [25]. Plant growth-promoting rhizobacteria (PGPR) are microorganisms that naturally occur in the area around or on the root surface. It has been observed that PGPR can induce plant resistance against herbivores and attract natural enemies [26,27]. Additionally, they contribute to the increase of glucosinolate content produced by the plants [28] and to the inhibition of the development of pathogenic microorganisms [29,30,31,32,33]. PGPRs potentially hold great importance for agricultural ecosystems to reduce the use of agrochemicals, such as fertilizers and pesticides [34,35]. Plants, in turn, act as mediators of the interactions that occur between PGPR and aboveground insects [26,27].

Most of the studies about aboveground multitrophic interactions do not consider interactions that occur in the soil system [36,37,38]. Beneficial soil-borne microorganisms can affect aboveground interactions in different ways, including promoting plant growth and improving plant development, and altering plant biochemical pathways and the VOCs blends released by plants, making them more attractive to natural enemies [39]. However, few studies have evaluated the mechanisms involved in symbiotic interactions over various trophic levels. Guerrieri et al. [19] verified that tomato plants in symbioses with the arbuscular mycorrhizal fungus, *Glomus mosseae* BEG 12, improved the attractiveness of the generalist aphid parasitoid *Aphidius ervi* (Hymenoptera: Aphidiidae). Tomato roots colonized with a non-mycorrhizal plant growth-promoting fungus, *Trichoderma longibrachiatum* strain MK1, were more attractive to aphid parasitoids and predators when compared with the uncolonized plants and showed quantitative differences in the release of specific volatile compounds [40]. Researchers have reported that rice plants inoculated with some strains of *Pseudomonas fluorescens* showed higher activity of polyphenol oxidase and lipoxygenase and that these inductions could be involved in enhanced performance by natural enemies of the rice leaf folder [41]. 

How soil-borne beneficial microorganisms, mainly PGPR, can interact and affect herbivorous natural enemies is a potential gold mine to be explored. The knowledge of the mechanisms involved in these interactions can be useful to improve integrated pest management (IPM) tactics. Studies have focused on exploring below- and aboveground interactions involving single species systems; however, these oversimplified systems are far from the naturally occurring interactions. An increase of the complexity of the systems is required to increase the reliability of the application of beneficial microbes in IPM (reviewed in [42]).

In this work, we studied how soil bacteria, plants, herbivores, and natural enemies interact through plant odors. To do so, an ecological system was composed of the following: A commercially produced PGPR [43], *Bacillus amyloliquefaciens* strain GB03 (previously described as *B. subtilis* GB03) [44]; arugula plants (*Eruca sativa*), a Brassicaceae; the diamondback moth, *Plutella xylostella* (Lepidoptera: Plutellidae), a crucifer specialist herbivore [45]; the fall armyworm *Spodoptera frugiperda* (Lepidoptera: Noctuidae), a generalist herbivore; and the earwig *Doru luteipes* (Dermaptera: Forficulidae), a generalist predator [46,47,48,49]. Then, we addressed the following questions: (i) Does the predatory earwig have any preference over odors emitted by arugula plants infested with generalist or specialist herbivores? and (ii) could that behavior be modified by inoculating plants with GB03? As a generalist predator, we hypothesized that *D. luteipes* (i) would be guided by the plant odors blends produced by the specialist over the generalist herbivore, and (ii) due to the symbiosis between the plant and PGPR, we also expected that earwigs would be attracted mostly to inoculated GB03 plants. We conducted a series of olfactometer assays during the scotophase to answer these questions. Our results help to understand how the symbiotic interactions that occur in a multitrophic system could be useful for improving biological control.

## 2. Materials and Methods

### 2.1. Bacterial Culture and Plant Treatments

*Bacillus amyloliquefaciens* GB03 was streaked onto Trypticase Soy Agar (TSA) plates and incubated at 28 °C in an incubator chamber shielded of light for 24 h. After, GB03 colonies were harvested from TSA plates into Falcon tubes with 5 mL of Trypticase Soy Broth (TSB) medium and were shaken overnight in a dark room at 28 °C and 150 rpm. The GB03 suspension concentration was determined by optical density (OD_600_ = 0.7) and adjusted to yield 10^9^ colony forming units (CFU) mL^−1^. 

Arugula seeds were immersed in a bacterial suspension culture or TSB medium for 30 min and sown in Basaplant^®^ (Base Agro, Artur Nogueira, Brazil), potting soil (250 mL) with 2.5 g of fertilizer (Osmocote Plus^®^ 14-14-14, ICL, Summerville, SC, USA). All plants were grown in a greenhouse under natural light and temperature from November 2017 to March 2018 in Piracicaba, São Paulo State, Brazil. They were watered three times a day with an automatic irrigation system until 30 days after emergence, after which they were used in the experiments.

### 2.2. Insect Rearing

#### 2.2.1. *Plutella Xylostella*

Caterpillars were provided from a stock rearing kept in the Laboratory of Chemical Ecology and Insect Behavior of Luiz de Queiroz College of Agriculture (ESALQ-USP, Piracicaba, Brazil). Moths were kept into an acrylic cage (30 × 40 × 30 cm) with a front opening (13 cm in diameter) covered by a thin cloth mesh. Arugula plants were supplied as oviposition substrate. After hatching, neonate caterpillars were transferred to plastic pots (5 × 15 × 12 cm). For caterpillars’ feeding, cabbage leaves (*Brassica oleracea*) were provided as a natural diet and changed daily until the caterpillars reached the pupal stage when they were transferred to the adult acrylic cage. The rearing was kept in a insect rearing chamber under controlled conditions (25 ± 3 °C, 65 ± 5% RH, 12 L:12 D). 

*Spodoptera frugiperda*: Caterpillars were reared under laboratory conditions (25 ± 3 °C, 60 ± 10% rh, 12 L:12 D) and fed an artificial diet as described by [49]. Larvae were placed in plastic containers (5 cm high × 5 cm diameter) on the diet. Pupae were separated according to the gender and adult couples were placed into polyvinyl chloride (PVC) cages (10 cm diam × 22 cm high) closed with Petri dishes (14.2 cm). As a surface for oviposition, cages were lined with white paper. Adults were fed 10% honey solution every 2 days. 

#### 2.2.2. *Doru Luteipes*

Females of the earwig were collected by hand in maize and sugar cane plantations in Piracicaba, Brazil. The individuals were taken to the laboratory and kept in closed plastic boxes (23 × 7 × 14 cm). *D. luteipes*’s natural habitat was simulated by adapting the methods described by [50]. Plastic boxes were covered with aluminum foil to reduce light incidence. To supplement the oviposition substrate and refuge areas, pieces of wet cotton were inserted into 3-cm-long sections of transparent drinking straws. Corrugated cardboard was placed in the boxes as additional refuge areas to reduce cannibalism. The earwigs were fed an artificial diet consisting of dry cat food, which contains animal and plant protein and fat, as well as various vitamins and minerals (35%), wheat bran (27%), brewer’s yeast (23%), milk powder (14%), methylparaben (0.5%), and sorbic acid (0.5%). The pieces of moistened cotton and food were replenished twice weekly. 

### 2.3. Plant Weight Measurements

Plants inoculated with the rhizobacteria GB03 grow larger and faster than non-inoculated plants [51]. Whereby, intending to observe if the inoculated plants acquired the rhizobacteria GB03, the difference between the fresh and dry leaf weights of inoculated and non-inoculated plants was evaluated. A total of 20 4-week-old plants (10 inoculated and 10 non-inoculated) were selected and removed from pots. Fresh and dry leaf weights were measured on an analytical scale. After fresh weight measurement, leaves were placed into an oven at 60 °C for 72 h and re-weighted to obtain the dry weight.

### 2.4. Y-Tube Olfactometer Experiments

**Figure 1:**
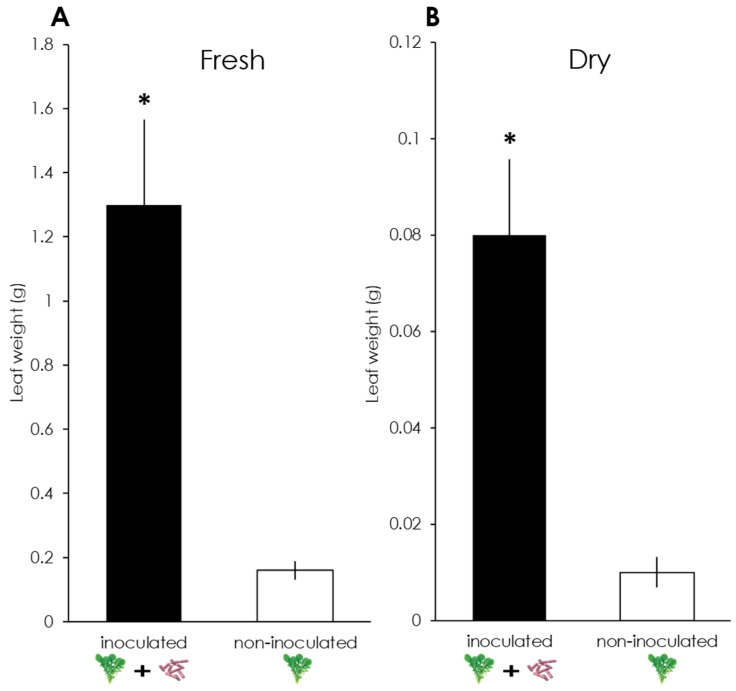
Fresh (**A**) and dry (**B**) leaf weights of *Eruca sativa* plants, inoculated and non-inoculated with *Bacillus amyloliquefaciens* GB03. *: *p* < 0.05 (Welch’s *t*-test). *n* =10.

One day prior to the experiments, inoculated and non-inoculated plants (30 days old) were transferred from the greenhouse to the laboratory and kept under supplementary lighting (60–80 µmol, 12 L:12 D). After, pots were covered with aluminum foil to avoid odors from the soil. Twelve hours prior to the experiments, five third-instar starved (24 h) caterpillars of *S. frugiperda* or *P. xylostella* were placed on arugula plants (*E. sativa*) to inflict herbivore damage and covered with voile bags (22 × 30 cm) for 12 h. We used 9 treatments, which we evaluated in 13 different combinations (see Figure 1): Non-inoculated undamaged (NU); inoculated *Plutella xylostella* (IP); non-inoculated *Plutella xylostella* (NP); inoculated *Spodoptera frugiperda* (IS); non-inoculated *Spodoptera frugiperda* (NS); inoculated undamaged (IU); Trypticase Soy Agar (TSA); and Trypticase Soy Agar and inoculum (TSA-GB03).

For the olfactometer experiments, female adult earwigs were used. Based on a previous experiment, we found that females are more responsive in the bioassays because they forage more than males since they use more energy producing eggs and taking care of the offspring [52,53]. Olfactometer assays were conducted at night (19:00–23:00 h) under controlled conditions (25 ± 1 °C, 70 ± 10% rh), following the methodology previously described by [54]. Clean and humidified air was supplied to the olfactometer system via an ARS Volatile Collection System (Gainesville, FL, USA). Clean air at a flow of 1 L/min was passed through each of two glass chambers (10 cm diam × 5 cm high), containing a single plant/treatment, and then into the two branches of the Y-tube olfactometer (main arm, 25 cm long; side arms: 20 cm long; 0.9-cm internal diameter). Earwigs were individually introduced into the long base of the Y-tube olfactometer and observed for 5 min or until a choice was made. A choice was defined when an earwig went beyond the halfway point of a branch. The individuals that did not choose a branch within 5 min were excluded from the statistical analysis. Each earwig was tested only once. When a clean Y-tube olfactometer was connected to the system, the treatments swapped branches and the Y-tube was rotated to avoid side bias. After being used once, each Y-tube olfactometer was washed with acetone (90% *v/v*) and dried at 170 °C for 2 min. After every 10 insects, a new pair of plants/treatments was used, totalizing 40 earwigs.

### 2.5. Statistical Analyses

The fresh and dry weights of inoculated and non-inoculated plants were compared using Welch’s *t*-test due to the heteroscedasticity of the data. We used the logistic regression and Wald’s Chi-square test to analyze the earwig choice in the olfactometer assays. Statistical analyses were performed using R version 3.5.1 (The R Foundation, Vienna, Austria).

## 3. Results

### 3.1. Plant Weight Measurement

A significant difference was observed in the fresh leaf weight between inoculated (M = 1.30 g, SD = 0.84) and non-inoculated plants (M = 0.16 g, SD = 0.09); t_(9.19)_ = 4.287, *p* = 0.002 (Figure 1A). There was also a significant difference in the dry leaf weight between inoculated (M = 0.08 g, SD = 0.05) and non-inoculated plants (M = 0.01 g, SD = 0.01); t_(9.22)_ = 4.522, *p* = 0.001 (Figure 1B).

### 3.2. Y-Tube Olfactometer Experiments

**Figure 2:**
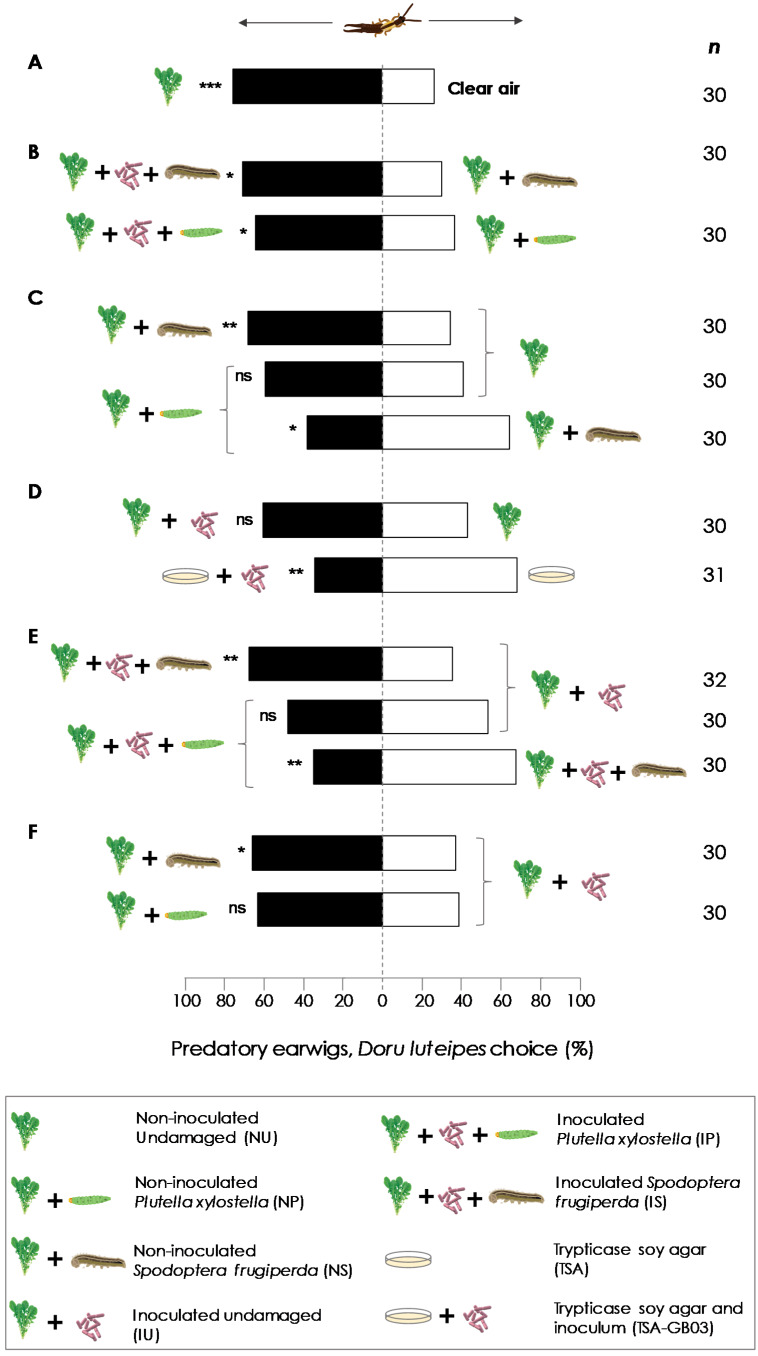
Olfactory responses of predatory earwigs, *Doru luteipes,* to arugula plants, *Eruca sativa*, and *Bacillus amyloliquefaciens* GB03 treatments (**A**–**F**) in a Y-tube olfactometer. Bars represent the overall percentages of earwig predators choosing each odor source. *: *p* < 0.05, **: *p* < 0.01, ***: *p* < 0.005, ns: no significant difference (for Wald’s Chi-square test), *n* is the number of responsive predators.

*Doru luteipes* females were responsive in olfactometer tests to *E. sativa* volatiles. They were significantly more likely to choose non-inoculated undamaged plants (NU) over clean air (CA) (*χ*^2^ = 13.59, df = 58, *p* < 0.001) (Figure 2A). More earwigs were more attracted by inoculated herbivore-damaged plant volatiles than non-inoculated undamaged plant volatiles, for both herbivores, *S. frugiperda* and *P. xylostella* (IS vs. NS: *χ*^2^ = 6.80, df = 58, *p* = 0.009; IP vs. NP: *χ*^2^ = 4.32, df = 58, *p* = 0.037, respectively) (Figure 2B).

When plants were non-inoculated, earwigs preferred the volatiles from damaged plants by *S. frugiperda* (NS) over NU volatiles (*χ*^2^ = 6.35, df = 58, *p* = 0.012). The attraction of earwigs to non-inoculated plants damaged by *P. xylostella* (NP) was less evident. Despite 60% of the earwigs being attracted by NP over NU, there were no statistically significant differences (*χ*^2^ = 2.42, df = 58, *p* = 0.120). When earwigs were exposed to non-inoculated plants that were herbivore damaged by both species of caterpillars (NS vs. NP), they preferred odors from NS (*χ*^2^ = 4.32, df = 58, *p* = 0.038) (Figure 2C). Earwigs did not show any preference to inoculated undamaged plants (IU) over NU (*χ*^2^ = 6.80, *p* = 0.009). Additionally, when they were exposed to odors from TSA and TSA containing GB03 inoculum, they preferred TSA only (Figure 2D).

Regarding the odors of plants with induced herbivore damage, the same behavior as described above was observed. Earwigs preferred inoculated plants damaged by *S. frugiperda* (IS) over non-induced IU (*χ*^2^ = 6.35, *p* = 0.012). There were no statistical differences when inoculated plants damaged by *P. xylostella* (IP) and IU were compared (*χ*^2^ = 0.601, *p* = 0.438). Nevertheless, when earwigs were exposed to IS and IP, they preferred IS (*χ*^2^ = 4.32, *p* = 0.011) (Figure 2E). Earwigs preferred odors from NS over IU (*χ*^2^ = 1.09, df = 58, *p* = 0.041). There were no statistical differences when NP was compared to IU (*χ*^2^ = 3.19, df = 60, *p* = 0.074), despite 61% of the earwigs being attracted by NP (Figure 2F). 

## 4. Discussion

In this study, we showed that the symbiotic interaction between GB03 and plants infested with herbivores may favor the attraction of predatory earwigs. Soil-borne microbial mutualists, such as rhizobacteria and mycorrhizal fungi, can affect plants in several ways. For example, they can promote growth and higher yields when used as a biofertilizer [55,56,57]. Here, we noticed significant differences in the fresh and dry leaves weights when plants were inoculated with GB03, meaning that the rhizobacterium colonized arugula roots. Previous research has shown that plants inoculated with GB03 grow larger and faster than non-inoculated plants, and that microbial VOCs, such as 2,3-butanediol and acetoin, might be responsible for the improved development [51]. Thus, the positive effect of GB03 on the arugula plants’ biomass increase shows the beneficial role of this PGPR and its potential application in agriculture. 

In the olfactometer bioassays, earwigs responded preferentially to odors emitted by TSA over TSA-GB03 volatiles. This could be due to the strong bacteria/rot smell released by the TSA with GB03, which deterred the earwigs. Since all of the plants in these experiments had the pot and soil covered by aluminum foil, VOCs released from the bacteria in the soil may not have made their way into the Y-tube olfactometer. Earwigs rarely fly, so it would be interesting to find out if they are deterred by odors from the soil when seeking out foraging sites and whether the odors produced by GB03 in the soil affect them in any way. In addition, bacteria growing on agar probably do not emit the same volatiles as they do in the soil or when they colonize the roots.

Previous research has found that *D. luteipes* is attracted to nocturnal HIPVs emitted by maize (Delprim) irrespective of whether herbivory was inflicted by a leaf chewer or a stem borer caterpillar [51]. Here, we showed that this earwig is also attracted by nocturnal odors emitted by arugula plants in different conditions (undamaged or herbivore damaged, inoculated or not inoculated by GB03). Instead of what was predicted, in the olfactometry assays, the earwigs were attracted to plant odors triggered by *S. frugiperda* but not by *P. xylostella*, whether the plants were inoculated or not inoculated with GB03. *Doru luteipes* has been recognized as a generalist predator of lepidopteran eggs and larvae in maize, soybean, sugarcane, and *B. oleracea* crops [47,48,49,58,59]. Natural occurrence and predation of *P. xylostella* by the earwig species *Euborellia annulipes* (Dermaptera: Anisolabididae) has been reported in kale crops and intercrops with *Lobularia maritima* (Brassicaceae) [60]. This lack of preference for *P. xylostella* could be due to the origin of the earwigs used in this study, as they were from maize and sugarcane plantations, where *S. frugiperda* is a big part of their diet. In the field, predatory arthropods coexist with a host plant and prey. Hence, they constantly experience HIPVs resulting in a positive association, leading the predators to avoid the risk of visiting a plant without prey [61]. This implies that although the earwig is a generalist predator, it has a prey preference. It is also important to highlight that *D. luteipes* and *S. frugiperda* have a long evolutionary history together and are frequently observed on crops [62]. However, we should not discard the possibility that the earwigs could be attracted to *P. xylostella*-induced plant volatiles after a short time of exposure to these HIPVs before the experiments, once it has been shown that predatory arthropods can learn to associate odors with the occurrence of prey [63,64,65].

When we compared undamaged plants inoculated with GB03 (IU) with non-inoculated plants damaged by *S. frugiperda* (NS) and *P. xylostella* (NP), earwigs were attracted only to the plants damaged by *S. frugiperda* (NI). It corroborates our previous statement about prey preference. On the other hand, it means that arugula HIPVs are more likely to have an important role in attracting *D. luteipes* than the constitutive odors released by plants that have only been inoculated with GB03. In maize, *D. luteipes* seems to use HIPVs comprised of mainly green leaf volatiles (GLV), such as (*E*)-2-hexenal, (*E*)-3-hexenal, (*Z*)-3-hexen-1-ol, and (*Z*)-3-hexenyl acetate, to find their preys [54]. Here, we did not characterize the chemical profile of arugula plant volatiles. However, the orientation of generalist natural enemies to GLVs seems to be a common strategy for prey searching, and arugula plants might also release some of these fatty acid derivatives upon herbivory. 

Nonetheless, our most relevant result was the synergistic effect of volatiles from inoculated and herbivore-damaged plants, in which the earwigs were attracted by the plants that presented both conditions for both specialist and generalist herbivores. In this case, the earwigs had no prey preference and were equally attracted to both. Thus, GB03 proved to have great value in our study system, especially in conserving natural enemies, such as earwigs. Ngumbi [66] showed that two species of parasitic wasps (Hymenoptera: Braconidae) with different degrees of host specificity, *Microplitis croceipes* (specialist) and *Cotesia marginiventris* (generalist), were more attracted to cotton plants that were treated with a suspension of bacilli strains over untreated plants. The author postulated that these results could be associated with the volatile organic compounds (VOCs) blend, which was presented in different compositions between PGPR-treated plants and untreated plants. The observed synergism may be due to a modification in the plant biochemistry caused by both inoculation and herbivory. Different studies have suggested that rhizobacteria benefit plants by altering their biochemistry against antagonist herbivores [25,67]. According to [68], GB03 VOCs trigger a metabolic pathway that requires ethylene, which has been reported to increase the volatile emission induced by (*Z*)-3-hexenol [68]. Ethylene precursors, when applied exogenously on lima bean plants, increased the jasmonic acid and induced HIPVs release, resulting in the attraction of the predatory mite, *Phytoseiulus persimilis* (Acari: Phytoseiidae) [69]. Studies with the application of mixtures of other rhizobacteria, such as *Pseudomonas fluorescens* strains in rice, led to less damage by leaf folder pests and greater natural enemy populations compared with untreated controls under greenhouse conditions. These results could be related to the highest activities of polyphenol oxidase (PPO) and lipoxygenase (LOX), which were used to treat the plants demonstrated during this study [41].

Previous research has focused on the effects of bacterial volatiles on plant growth and induced systemic resistance towards plant pathogens (e.g., [70]), while there are few studies of VOCs production by soil bacterial and fungal mutualists of plants with indirect plant defenses [71]. Other than mediating the indirect defense by attracting natural enemies, GB03 can also induce direct defense mechanisms. GB03-inoculated *Arabidopsis* enhanced sulfur metabolism in response to herbivory [28]. Here, we highlight that plant–GB03 symbiosis offers a remarkable potential for sustainable agriculture, through the attraction of a generalist predator in a *Brassicaceae* crop. 

## 5. Conclusions

Our study revealed that the interaction between rhizobacteria, plants, and herbivores can affect the predatory earwig’s behavior. A synergistic effect of PGPR-inoculated and herbivore-damaged plants resulted in earwigs being attracted by the plants that presented both conditions for both specialist and generalist herbivores. This positive interaction of PGPR and indirect plant defense enhances the benefits of the use of plant-associated microbes in agriculture. Our findings help to fill the important knowledge gap regarding multitrophic interactions and should provide useful guidelines for their application to agricultural fields. Further studies are required to investigate which chemical and physiological mechanisms are involved in these interactions. Additional studies should be done to test the efficiency of the PGPR inoculation in the attraction of natural enemies in other systems and how PGPR could be employed in field crops to contribute to IPM.
